# *In vitro* study of ethanol production, ethanol tolerance, and antimicrobial susceptibility of gut microbes associated with liver diseases

**DOI:** 10.1080/29933935.2026.2664983

**Published:** 2026-04-29

**Authors:** Anissa Idrissa Abdoulaye, Babacar Mbaye, Reham Magdy Wasfy, Louis Carmarans, Mamadou Beye, Claudia Andrieu, Sofiane Bakour, Aïcha Hamieh, Nicholas Armstrong, Patrick Borentain, Fadi Bittar, Stéphane Ranque, Jean-Marc Rolain, Gregory Dubourg, Jean-Christophe Lagier, Maryam Tidjani Alou, René Gerolami, Matthieu Million

**Affiliations:** a Aix Marseille Univ, MEPHI, Marseille, France; b IHU-Méditerranée Infection, Marseille, France; c Assistance Publique Hôpitaux de Marseille (APHM), Marseille, France; d Unité Hépatologie, Hôpital de la Timone, APHM, Marseille, France; e Aix Marseille Université, APHM, SSA, RITMES, Marseille, France

**Keywords:** Liver diseases, metabolic dysfunction-associated steatohepatitis (MASH), alcoholic hepatitis (AH), endogenous ethanol production, gut microbiota-targeted approach

## Abstract

Endogenous ethanol (EtOH) production is a newly identified pathophysiological mechanism involved in metabolic dysfunction-associated steatohepatitis (MASH) and liver disease associated with hepatitis B virus (HBV). Therefore, the characterization of EtOH-producing species associated with liver disease could contribute to the development of gut microbiota-targeted approaches. We investigated EtOH production and tolerance, antimicrobial susceptibility and antimicrobial resistance gene(s) in 33 strains isolated in previous culturomics studies and belonging to species enriched in MASH, alcoholic hepatitis (AH) and HBV patients. *Enterocloster clostridioformis*, *Thomasclavelia ramosa* and *Peptinophilus grossensis* were identified as new EtOH-producing species associated with liver diseases. A strong association between EtOH tolerance and production was detected (*p* < 0.05). Yeast, *Enterocloster* species (strictly anaerobic bacteria) and *Limosilactobacillus fermentum* produced the highest concentrations of EtOH (0.8 to 3.3 g/L). The poorly absorbed drugs, amphotericin B, rifaximin and vancomycin together showed high *in vitro* susceptibility. Furthermore, *E. clostridioformis* EC38 harbored the *vanB* operon. New EtOH-producing species associated with liver diseases were identified thanks to culturomics. Notably, most of them are anaerobic bacteria. These findings underscore the need to further investigate anaerobic gut microbiota species enriched in liver diseases with the aim of developing gut microbiota-targeted therapies.

## Graphical abstract

**Figure:**
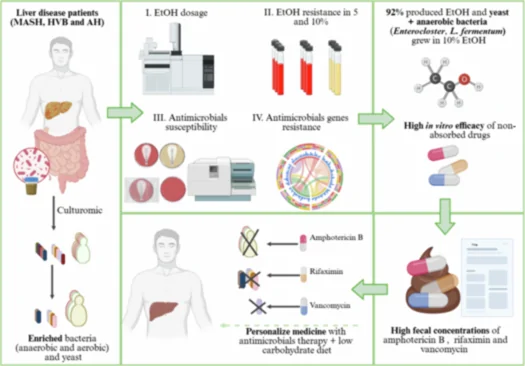

## Introdction

Liver diseases are a major public health problem. A study estimated that, in 2023, more than 2 million deaths, or 4% of the global mortality rate, are annually caused by cirrhosis, viral hepatitis and liver cancer.^1^ The main causes of cirrhosis are chronic infection with hepatitis B virus (HBV) or hepatitis C virus (HCV), alcoholic hepatitis (AH), and metabolic dysfunction-associated steatohepatitis (MASH).^2^ The incidence of metabolic hepatic diseases, including metabolic dysfunction-associated fatty liver disease (MAFLD) and its severe form, MASH, is growing worldwide. Indeed, in 2019, the estimated prevalence of MAFLD was 1.6 billion worldwide.^3^ It increased from 25% between 1990 and 2006 to 38% between 2016 and 2019.^4^ The global prevalence of MASH is estimated to be 5%, with the highest prevalence in Latin America (44%).^4^

Alterations in the gut microbiota have been observed in viral hepatitis,^5^ ^,^ ^6^ AH,^7^ MAFLD and MASH.^8^ Moreover, one feature of MASH is endogenous ethanol production,^9^ which was initially demonstrated by Yuan et al. with *Klebsiella pneumoniae* strains isolated from patients with auto-brewing syndrome (ABS) and MASH.^10^ Similarly, Meijnikman et al. reported a positive correlation between abundance of *Lactobacillaceae* and endogenous ethanol production in MAFLD patients.^11^ Interestingly, the causal role of these microbes in endogenous ethanol production was confirmed through *in vitro* and *in vivo* experimental studies.^10^ ^,^ ^12^

The instrumental role of the gut microbiota has been further supported by studies showing that antimicrobial agents can improve the prognoses of certain liver diseases. In particular, a randomized, controlled trial (RCT) showed that rifaximin improved liver markers and insulin resistance in patients with MASH.^13^ Nevertheless, it should be noted that this study did not provide information regarding modifications of gut microbiota. In humans, only one study has used antimicrobials targeting ethanol-producing microbial strains, *Klebsiella pneumoniae,* with promising results.^10^ Accordingly, we hypothesized that identifying candidate instrumental microbes producing ethanol is an essential preliminary step before any interventional studies can be performed. To our knowledge, only three studies have identified five species associated with liver disease and confirmed *in vitro* ethanol production from patient strains: *K. pneumoniae* ^10^; the three yeasts *Pichia kudriavzevii, Candidaalbicans*, and *Nakaseomyces glabratus* ^14^; and *Enterocloster bolteae.* ^15^ Therefore, the specific aim of the present study was to identify whether other microbial species associated with liver disease, strains of which were isolated from patients, could produce high ethanol levels. To do so, we selected strains corresponding to species enriched in liver disease in published studies and isolated from patients with liver diseases (MASH, AH and HBV) included in our HEPATGUT study.^14-17^ We subsequently tested each of these strains for ethanol production, ethanol resistance, antibiotic susceptibility and antimicrobial resistance genes by genome sequencing. We tested all antibiotics routinely tested in our clinical microbiology laboratory but focused particularly on non-absorbable antibiotics, notably those already used in patients, such as rifaximin,^13^ vancomycin^18^ and amphotericin B.^19^

## Materials and methods

### Ethical approval

All the strains used in this study were isolated in the HEPATGUT project, which was approved by the ethics committee and Comité de Protection des Personnes (CPP: 21.04391.000046-21075). The Ile de France XI Human CPP issued a favorable opinion on January 4, 2022. The members of this committee are Ariane QUEFFELEC, Axel LEVIER, Delphine REGNAULT-ROGER, Annie DURAND, Caty EBEL BITOUN, Sabine de la PORTE, Gérard LOEB, Kolia MILOJEVIC, Léon LOISEAU, Nicole TAVERNY, Olivier LANTRES, Michèle CATZ, Odile LACHAUD, and Jean-François LAIGNEAU. This study was conducted in accordance with the Declaration of Helsinki (World Medical Association, 2024), and informed consent (written information and verbal nonopposition in accordance with French regulations) was obtained from each participant.

### Studied strains

We studied strains enriched in patients with liver disease in published case‒control studies that had available isolates from our previous culturomics studies.^14^ ^,^ ^16^ ^,^ ^17^ The associations of these species with liver disease were demonstrated either by culturomics (difference in frequency) or by 16S rRNA gene amplicon sequencing targeting the V3-V4 region (difference in frequency or differential abundance). A total of thirty-three strains, including bacteria and yeasts, were selected from culturomics case‒control studies of patients with MASH, HBV, and AH.

Strains of species enriched in patients and those enriched in controls were studied regardless of the origin of the sample (Table 1). Hence, we included the following species enriched in the intestinal microbiota of MASH patients: *Bacteroides thetaiotaomicron*, *C. albicans, E. bolteae*, *Enterocloster clostridioformis*, *K. pneumoniae*, *Limosilactobacillus fermentum*, *Mediterraneibacter gnavus*, *N. glabratus*, *Peptoniphilus grossensis, P. kudriavzevii* and *Thomasclavelia ramosa.* ^14^ ^,^ ^16^ Strains of *T. ramosa* enriched in the gut microbiota of patients with AH and chronic HBV infection have also been studied.^17^ A strain of *Klebsiella michiganensis* that was identified as *Klebsiella oxytoca* by MALDI‒TOF MS prior to sequencing was also selected. This choice was made because *K. oxytoca* is phylogenetically close to *K. pneumoniae.* Moreover, we previously identified a high ethanol-producing *K. oxytoca* strain from a MASH patient.^14^

**Table 1.** Strains tested.

Genus/Species	Strain (Abbreviation)	CSUR N°	Control	MASH	Alcoholic hepatitis	Chronic HBV infection
**Strains of species** **enriched in controls**						
*Alistipes shahii*	*Alistipes shahii T1 (AST1)*	QA2153	*			
	*Alistipes shahii S4 (ASS4)*	QA2154		*		
*Bacteroides uniformis*	*Bacteroides uniformis S4 (BUS4)*	QA2155		*		
**Strains of species** **enriched in liver diseases**						
*Bacteroides thetaiotaomicron*	*Bacteroides thetaiotaomicron LF 115 (BTLF)*	Q9811	*			
	*Bacteroides thetaiotaomicron S7 (BTS7)*	QA0002		*		
	*Bacteroides thetaiotaomicron S4 (BTS4)*	QA0001		*		
	*Bacteroides thetaiotaomicron N3 (BTN3)*	Q9577		*		
*Candida albicans*	*Candida albicans N10 (CA10)*	L0406		*		
	*Candida albicans N6 (CA6)*	L0407		*		
	*Candida albicans O59 HF (CA059)*	L0417		*		
*Enterocloster* spp.	*Enterocloster bolteae 42 S1R (EB42)*	QA0130		*		
	*Enterocloster bolteae 1' S8 R (EB1)*	Q9761		*		
	*Enterocloster clostridioformis 38’ S1R (EC38)*	QA0131		*		
	*Enterocloster clostridioformis S8 39 (EC39)*	Q9580		*		
*Klebsiella* spp.	*Klebsiella michiganensis N7 (KMN7)*	Q9571		*		
	*Klebsiella pneumoniae N7 (KPN7)*	Q7093		*		
	*Klebsiella pneumoniae S4 (KPS4)*	Q9556		*		
	*Klebsiella pneumoniae S6 (KPS6)*	Q7092		*		
*Limosilactobacillus fermentum*	*Limosilactobacillus fermentum 46 S8-S9R (LF46)*	Q9857		*		
*Mediterraneibacter gnavus*	*Mediterraneibacter gnavus 14 S8 R (MG14)*	Q9760		*		
*Nakaseomyces glabratus*	*Nakaseomyces glabratus N1 (NG1)*	L0411		*		
	*Nakaseomyces glabratus O59HF (NGO59)*	L0418		*		
*Peptoniphilus grossensis*	*Peptoniphilus grossensis 58’ S1R (Pgro)*	QA0129		*		
*Pichia kudriavzevii*	*Pichia kudriavzevii N2 (PKN2)*	L4010		*		
	*Pichia kudriavzevii N3 (PKN3)*	L0409		*		
	*Pichia kudriavzevii N5 (PKN5)*	L4012		*		
	*Pichia kudriavzevii Nash8 (PKN8)*	L0408		*		
*Thomasclavelia ramosa*	*Thomasclavelia ramosa S3R (TRS3)*	QA0666			*	
	*Thomasclavelia ramosa 12' S8 (TR12)*	Q9759		*		
	*Thomasclavelia ramosa O85 LT (TRO85)*	Q9779	*			
	*Thomasclavelia ramosa O59 (TRO59)*	Q9705		*		
	*Thomasclavelia ramosa S5R (TRS5)*	Q9850			*	
	*Thomasclavelia ramosa S20R (TRS20)*	Q9849				*
	*Thomasclavelia ramosa S39R (TRS39)*	QA0118			*	
	*Thomasclavelia ramosa S15R (TRS15)*	QA0117			*	
	*Thomasclavelia ramosa S1R (TRS1)*	QA0481		*		

CSUR N**°**: Number in Collection de souche de l'unité des Rickettsies, MASH: metabolic dysfunction-associated steatohepatitis, HBV: hepatitis B virus, *: Strains origin.

In addition, species significantly depleted in the intestinal microbiota of MASH patients compared with healthy individuals were included, specifically *Alistipes shahii* and *Bacteroides uniformis.* ^16^ In fact, *A. shahii* is known to have beneficial effects on various pathologies, including liver fibrosis.^20^ Moreover, *B. uniformis* is a known gut commensal that has been shown to alleviate MASH.^21^

### *In vitro* ethanol production assay

For each species, ethanol production was quantified in triplicate in the initial isolation medium. The quantification of ethanol production by bacteria was carried out by inoculating 250 µL of 0.5 McFarland for aerobic bacteria and 1 McFarland for anaerobic bacteria in 5 mL of Columbia broth base enriched with 5% defibrinated sheep blood (COS) at 37 °C for 24 hours for aerobic bacteria and 48 hours for anaerobic bacteria. The quantification of ethanol production by yeasts was carried out by inoculating 250 µL of 0.5 McFarland in 5 mL of Sabouraud broth (Oxoid, Basingstoke, UK) at 30 °C for 24 hours. One milliliter of each culture and a negative control (sterile culture medium) were subsequently transferred to 20 mL glass headspace vials (Supplementary materials and methods).

Considering the difference in glucose concentration between the COS (5 g/L) used for bacteria and the Sabouraud broth (20 g/L) used for yeast, the highest concentration of ethanol-producing bacterial strains was also tested using modified COS broth (COS with 20 g/L glucose).

The ethanol concentration was determined using a headspace gas chromatography‒mass spectrometry (HS‒GC‒MS) system (Perkin Elmer, Villebon sur Yvette, France) in the Swafer D7 setup, which included an HS110 headspace injector, a Clarus 690 gas chromatograph and an SQ8T mass spectrometer, as described by Sissoko et al.^22^.

The timepoints of 24 h for aerobic bacteria and yeast and 48 h for anaerobic bacteria, as implemented in this study, align with their respective stationary phases.^23-25^ Uniform timepoints were used throughout all the experiments, as the objective of this research was to assess every characteristic under consistent conditions.

The limit of detection of HS-GC‒MS is less than 0.25 mM, while the limit of quantification is 0.25 mM. The retention time %RSD (relative standard deviation) was less than 0.1% after 10 repeated injections (*n* = 10), and the %RSD for analysis (including preparation and injection) was less than 7% (*n* = 10). The method demonstrates linearity within the range of 0.25–100 mM or 0.1–5% (17–900 mM). Ethanol quantification is based on this calibration curve using internal calibration with isopropanol, according to the equation Y = ax + b, where Y represents mM isopropanol × (Area_ethanol_/Area_isopropanol_), and X denotes mM_ethanol_. This method involves the detection of different alcohol structures (methanol, ethanol, isopropanol, and butanol) depending on different retention times and different monitored mass fragments.

### Ethanol tolerance test

The ethanol tolerance of bacteria was assessed in COS broth containing 0%, 5% and 10% ethanol, whereas that of yeast was assessed in Sabouraud broth (Oxoid) supplemented with 0%, 5% and 10% ethanol (Supplementary materials and methods). A strain was considered ethanol tolerant when at least one colony grew in the presence of a specific concentration of ethanol (5% or 10%) in the medium.

### Statistics

To compare quantitative variables, the Mann‒Whitney test or Student’s t test was applied on the basis of the data distribution. All tests were two-tailed. A *p* value < 0.05 was considered to indicate statistical significance. All the statistical analyzes were performed using GraphPad Prism software, version 10.4.1 (GraphPad Software, Boston, Massachusetts, USA; www.graphpad.com).

### Antifungal and antibiotic susceptibility tests

Antifungal susceptibility testing to amphotericin B, caspofungin, flucytosine, micafungin and voriconazole was conducted using the VITEK 2 automated system (bioMérieux, Marcy l'Etoile, France) and VITEK 2 AST-YS08 antifungal cards (bioMérieux, Durham, NC, USA). Susceptibility to fluconazole was assessed using E-test strips (bioMerieux, Marcy l'Etoile, France) (Supplementary materials and methods).

Susceptibility to 25 antibiotics was assessed with the disc diffusion method using E-test strips (bioMerieux) (Supplementary materials and methods). Considering the widespread use of rifaximin in the treatment of patients with liver disease, we also assessed susceptibility to rifaximin. The minimum inhibitory concentration (MIC) of rifaximin for each strain was assessed using the agar dilution method, according to the recommendations of the Clinical Laboratory Standards Institute (CLSI), as there were no E-test strips commercially available for this molecule.

### Ratio of antimicrobials’ fecal concentration on *in vitro* susceptibility results

In this exploratory study, we used a quantitative parameter to compare the reported fecal concentrations (FCs) of the least absorbable molecules (amphotericin B, rifaximin, and vancomycin)^26-28^ with the MICs obtained for our strains. An empirical criterion (FC/MIC >10) was applied for poorly absorbed antimicrobials to identify candidates for further gut-microbiota targeted approach studies. The resulting FC/MIC ratio was calculated as an exploratory indicator, based on the rationale that a ratio >10 has been used for aminoglycosides in serum (Maximal concentration (Cmax)/MIC >10).^29^ This threshold was applied conservatively across species, solely to highlight antimicrobials for potential further investigation, not as a clinical efficacy threshold.

### Antimicrobial resistance gene(s)

After bacterial genome sequencing and assembly (Supplementary materials and methods), the presence of antimicrobial resistance gene(s) was assessed by the command abritAMR with AMRFinder Plus via Galaxy Australia (https://usegalaxy.org.au/).

Additionally, for strains presenting high resistance to rifampin and rifaximin (>256 µg/mL), the presence of mutation(s) in the rifampin resistance-determining region (RRDR) in the *β* subunit of bacterial RNA polymerase (rpoB), which is the main reported cause of resistance to rifampicin,^30^ was assessed after genome annotation with PROKKA via Galaxy Australia (https://usegalaxy.org.au/, last accessed on October 16^th^, 2025) and *rpoB* alignment in MEGA 12 software (version 12.0.14).

Finally, in order to screen and characterize the vancomycin resistance genes identified for *E. clostridioformis* EC38 and EC39, the two strains were further sequenced with the Oxford Nanopore Technologies platform (Supplementary materials and methods). The presence of genes belonging to a vancomycin operon was verified according to Stogios et al.^31^ using AMRFinder Plus results. When gene-conferring resistance was present, alignment of the nucleotide sequences (blastn) using the National Center of Biotechnology Information (NCBI, https://blast.ncbi.nlm.nih.gov/) was generated between *Enterococcus faecium* strain SAU28 vancomycin resistance operon, complete sequence (KF823969.1) and *E. clotridioformis* genome.

## Results

### High ethanol production by microbial species associated with liver diseases

The production of ethanol by strains of species enriched in the intestinal microbiota of the controls (*A. shahii* and *B. uniformis*) was low, always less than 0.03 g/L (Supplementary Table 1 (Table S1)), compared with that of species enriched in the intestinal microbiota of liver disease patients (*n* = number of strains, median = median of ethanol dosed in g/L [interquartile range], *n* = 33, 0.58 [0.21–1.86] vs. *n* = 3, 0.026 [0.025–0.028], 22.3-fold, two-tailed Mann‒Whitney test, *p* < 0.0001; Supplementary Figure 1 (Figure S1)). Indeed, ethanol production was 22-fold greater in species enriched in the intestinal microbiota of patients with liver diseases than in those enriched in the intestinal microbiota of controls. A total of 92% (11/12) of the species associated with liver diseases produced detectable levels of ethanol, whereas none of the strains enriched in the controls (0/3) produced detectable levels of ethanol. *B. thetaiotaomicron* was the only species (1/12) enriched in the microbiota of patients with liver diseases that did not produce any detectable amount of ethanol.

**Figure 1.**
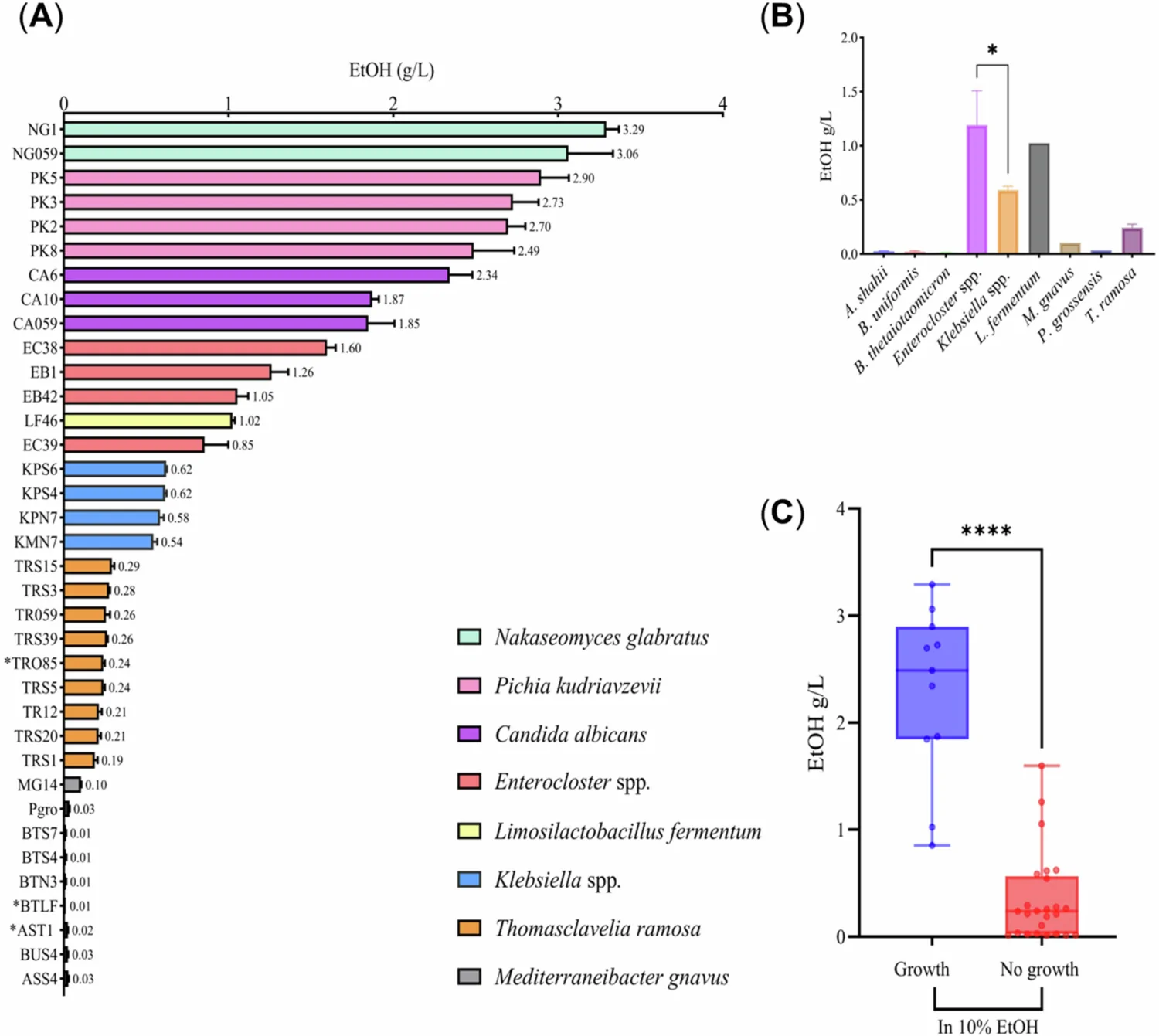
Ethanol production by yeast and bacteria dosed by GC‒MS and their ethanol tolerance. (A) NG: *N. glabratus*, CA: *C. albicans*, PK: *P. kudriavzevii*, KM: *K. michiganensis*, KP: *K. pneumoniae*, BU: *B. uniformis*, AS: *A. shahii*, BT: *B. thetaiotaimicron*, MG: *M. gnavus*, LF: *L. fermentum*, Pgro: *P. grossensis*, EB: *E. bolteae*, EC: *E. clostridioformis*, TR: *T. ramosa*, EtOH: Ethanol quantity dosed, *: Strains isolated from controls. (B) Ethanol production by species (EtOH: ethanol produced dosed). Level of significance: *: *p* = 0.0286 (two-tailed Mann–Whitney test). (C) Comparison of strains that grew in 10% ethanol medium with those that did not grow in terms of ethanol production (EtOH: quantity of ethanol dosed). Levels of significance: ****: *p* < 0.0001 (two-tailed Mann–Whitney test).

Analysis of the amount of ethanol produced by each strain revealed that, compared with the bacterial strains, the yeast strains produced 11 times more ethanol (*n* = 9, 2.70 [2.11–2.98] vs. *n* = 27, 0.24 [0.03–0.61], 11.25-fold, two-tailed Mann–Whitney test; *p* < 0.0001; Figure S2). The two strains of *N. glabratus* produced the greatest amount of ethanol (3.29 g/L and 3.06 g/L), followed by the *P. kudriavzevii* strains (2.89 g/L–2.48 g/L) and the *C. albicans* strains (2.34 g/L–1.84 g/L) (Figure 1A).

The two *E. bolteae* strains produced ethanol concentrations greater than 1 g/L (1.26 g/L and 1.05 g/L), as did one of the two *E. clostridioformis* strains (1.59 g/L and 0.85 g/L). *L. fermentum* produced 1.02 g/L ethanol. All the strains of *Klebsiella* spp. produced ethanol at concentrations slightly greater than 0.5 g/L (0.54–0.62 g/L). *T. ramosa* strains produced ethanol concentrations ranging from 0.18 g/L to 0.29 g/L. *M. gnavus* (MG14) and *P. grossensis* (Pgro) produced 0.10 g/L and 0.03 g/L ethanol, respectively. *B. thetaiotaomicron* strains produced the lowest concentration (0.01 g/L) (Figure 1A). Notably, strains from the same species produced similar amounts of ethanol, suggesting an overall species-dependent effect (Figure 1A). *Enterocloster* spp. produced twice as much ethanol as *Klebsiella* spp. did (Figure 1B) (*n* = 4, 1.16 [0.90–1.51] vs *n* = 4, 0.59 [0.55–0.62]; 1.97-fold, two-tailed Mann–Whitney test; *p* = 0.0286). The ethanol concentration measured in sterile media was consistently less than 0.006 g/L (Table S2).

As Sabouraud broth, which is used for yeast culture, contains 20 g/L glucose, we also assessed the ethanol production of the highest bacterial ethanol producers of each species in modified COS broth with 20 g/L glucose, namely *B. thetaiotaomicron* (BTN3), *E. clostridioformis* (EC38), *K. pneumoniae* (KPS6), *L. fermentum* (LF46) and *T. ramosa* (TRS5; Figure S3A). The increased glucose concentration resulted in increased ethanol production in some species (*L. fermentum* (LF46) and *K. pneumoniae* (KPS6)). Unexpectedly, the increased concentration resulted in decreased ethanol production in other species (*E. clostridioformis* (EC38) and *T. ramosa* (TRS5)) (Figure S3A). A comparison of ethanol production between bacterial and yeast strains in the presence of the same amount of glucose in the medium (20 g/L) was performed (Figure S3.B), revealing that, compared with bacteria, yeast still produced significantly more ethanol (*n* = 3, 2.8 [2.40–3.25] vs. *n* = 5, 0.58 [0.12–0.96], 4.8-fold, two-tailed Mann‒Whitney test; *p* < 0.0001). At 20 g/L, the same gradient of ethanol production per species was maintained, except for *E. clostridioformis* (EC38), which produced less ethanol than *K. pneumoniae* (KPS6) did (Figure S3.B).

### High ethanol production is associated with high ethanol tolerance

All the yeast strains grew in 10% ethanol, as did two bacterial strains, *L. fermentum* (LF46) and *E. clostridioformis* (EC39) (Table S3). All the bacterial strains grew at a concentration of 5% ethanol, except for *E. bolteae* (EB42) and *A. shahii* (ASS4) (Table S3). None of the species enriched in the intestinal microbiota of the controls grew in the presence of 10% ethanol (Table S3). The strains that grew in 10% ethanol produced significantly more ethanol (*p* < 0.0001) than those that did not (Figure 1C). The strains that were tolerant to 10% ethanol produced ten times more ethanol than those that were not (*n* = strains, m = median ethanol dose in g/L [interquartile range], *n* = 11, 2.49 [1.85–2.90] vs. *n* = 25, 0.24 [0.03–0.56], 10.4-fold, two-tailed Mann‒Whitney test, *p* < 0.0001; Figure 1C).

### High gut concentrations of amphotericin B, rifaximin and vancomycin compared to *in vitro* susceptibility results

**Figure 2.**
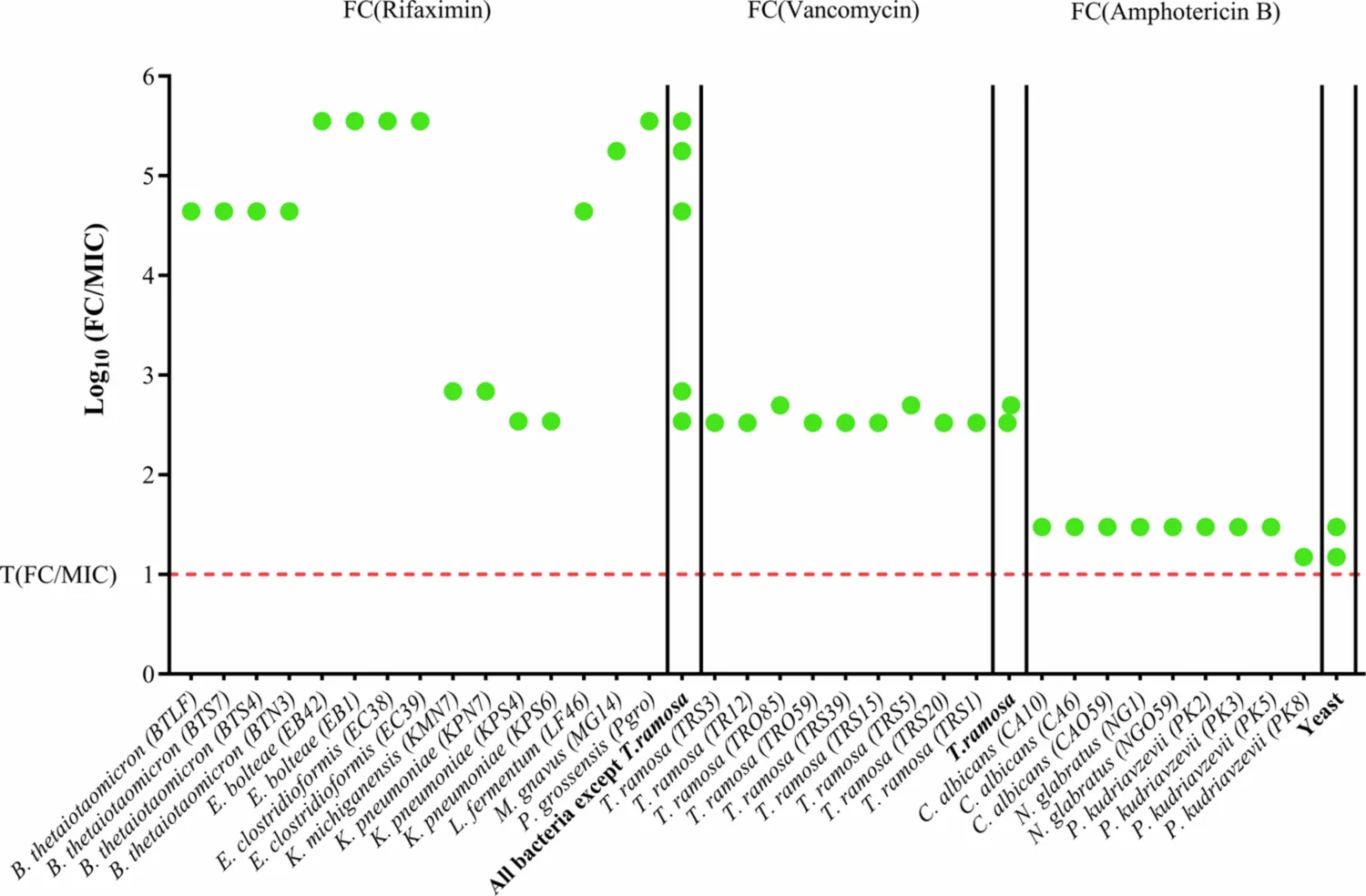
Potential *in vivo* efficacy of rifaximin, vancomycin and amphotericin B. Ratio of the fecal concentration after the recommended dose of amphotericin B, rifaximin and vancomycin to the MIC obtained after antifungal and antibiotic susceptibility tests. FC: Fecal concentration, MIC: Minimal inhibitory concentration, T: Threshold.

Based on the antifungal susceptibility results, amphotericin B demonstrated *in vitro* susceptibility against yeasts (Table S4). Its fecal concentration for the treatment of digestive candidiasis (2 g/day) was estimated at 60 μg/g of stool,^26^ yielding an FC/MIC ratio > 1 log10 (15- to 30-fold; Table S6, Figure 2).

For most bacteria, rifaximin displayed high *in vitro* susceptibility (Table S5). The fecal concentration of rifaximin used to prevent relapses in encephalopathy (1100 mg/day) is estimated at 11,000 μg/g of stool.^27^ Accordingly, the FC/MIC ratio ranged from 2 to 5 log10 (347- to 355,000-fold; Table S6). This ratio was highly variable depending on genus and species: 2–3 log10 for *K. pneumoniae*; 4–5 log10 for *B. thetaiotaomicron* and *L. fermentum*; and >5 log10 for all *Enterocloster* strains, *M. gnavus* and *P. grossensis* (Figure 2).

However, this ratio could not be estimated for *T. ramosa*, as its MIC was >256 µg/mL. Therefore, among poorly absorbed drugs, vancomycin showed *in vitro* susceptibility against *T. ramosa*. Because *T. ramosa* was previously known as *Clostridium ramosum* before reclassification, we used the fecal concentration of vancomycin based on the recommended posology for *Clostridioides difficile* infection (500 mg/day).^28^ The FC was estimated at 1000 μg/g of stool, resulting in an FC/MIC ratio >2 log10 (333- to 500-fold higher than the MICs) for all nine *T. ramosa* strains (Figure 2, Table S6). Notably, all these ratios were at least 15-fold higher than the MICs obtained.

### The detection of genes conferring resistance to vancomycin in *E. clostridioformis* aligns with the phenotypic profile

The assessment of resistance genes to rifaximin and vancomycin is crucial before the implementation of antibiotic therapy. Using AMRFinder Plus, which detects all resistance genes present in the NCBI database with at least 90% identity and containing gene *arr* alleles, we did not identify any *arr* genes in the bacterial genomes examined (Table S7). *T. ramosa* strains were the only ones with high resistance to rifampicin and rifaximin (>256 µg/mL; Table S5). Owing to the lack of susceptible *T. ramosa* strains and the important phylogenetic distance between *T. ramosa* and the main species studied in this context (*M. tuberculosis, E. coli* or *S. aureus*),^30^ the mapping of *rpoB* regions was performed with the *T. ramosa* reference genome (ASM1672878v1). This mapping revealed high similarity between our genomes and the reference one (only two mutations), with no common mutations found in the RRDR region (Table S8).

However, vancomycin resistance genes were detected in the genomes of *E. clostridioformis*. Specifically, strains EC38 and EC39 possessed three and ten vancomycin resistance genes, respectively (Table S7). EC38 genes belong to the operon *vanD*, whereas EC39 genes belong to the operons *vanD* and *vanB*. Both present the same genes for the operon *vanD* (*vanR-D*, *vanS-D* and *vanX-D*). *vanR-D*, *vanS-D*, and *vanX-D* are accessory genes (regulators and sensors) and are not directly involved in vancomycin resistance.^32^ Strikingly, EC39 also harbors all seven genes (*vanB*, *vanH-B*, *vanR-B*, *vanS-B*, *vanW-B*, *vanX-B*, *vanY-B*) of the *vanB* operon.^31^ To further verify the completeness of the operon *vanB*, an alignment in NCBI (blastn) among the *E. faecium* strain SAU28 vancomycin resistance operon, complete sequence (KF823969.1) and the genome EC39 was performed. The alignment results revealed a query coverage of 100% (1,029 bp) and 99.81% identity (Figure S4).

These results are consistent with the *in vitro* vancomycin susceptibility profile observed for *E. clostridioformis* EC39 (MIC 32 mg/L) and EC38 (0.38 mg/L), and for *E. bolteae* (EB1 0.50 mg/L and EB42 0.094 mg/L, Table S5) . Resistance genes against macrolides, streptogramin, lincosamide and tetracycline were also important genes within the studied genomes (Table S7).

## Discussion

**Figure 3.**
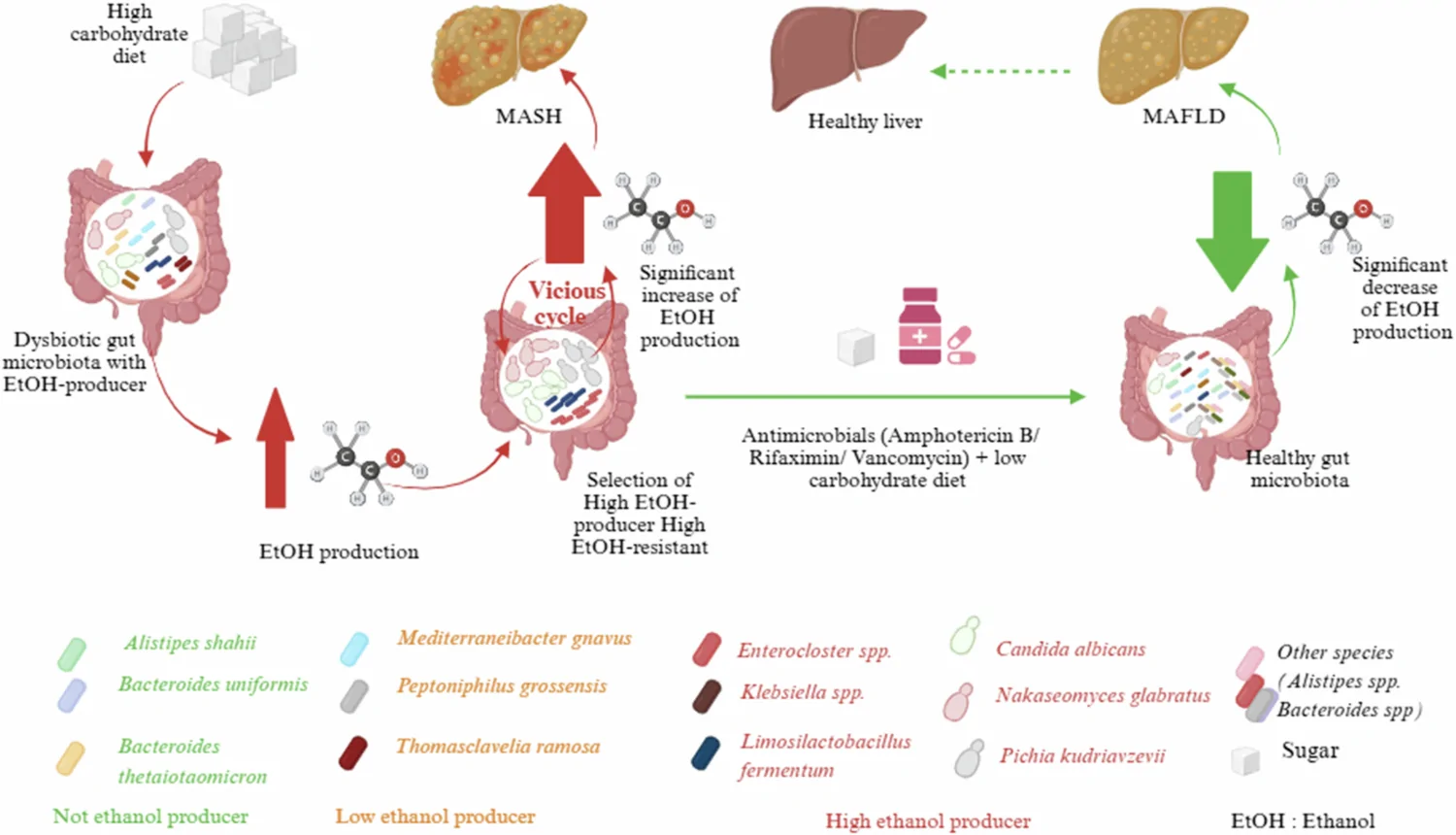
A vicious cycle caused by ethanol-producing ethanol-tolerant species is caused by the maintenance of a high-glucose diet and the potential utilization of a low-carbohydrate diet combined with antimicrobials as a gut microbiota-targeted to disrupt the cycle.

The increasing incidence of liver diseases and the instrumental role of gut dysbiosis in their pathogenesis highlight the need for improved therapeutic approaches. Therefore, in this study, we characterized species enriched in these pathologies and assessed their ethanol production and tolerance, as well as their antimicrobial susceptibility. We found that gut microbial species enriched in the intestinal microbiota of patients with liver diseases produced significant quantities of ethanol *in vitro*. Notably, most of them were capable of both producing ethanol and demonstrating ethanol tolerance, as reported by Yuan et al.^10^. Previous studies have reported ethanol production by the yeasts^14^ *K. pneumoniae,* ^10^ *E. bolteae,* ^15^ *L. fermentum* ^32^ and *M. gnavus.* ^33^ Ethanol tolerance has been reported in yeasts and *L. fermentum* at concentrations exceeding 10%.^34-37^ These findings indicate a potentially vicious cycle in diseases such as ABS and MASH when a high-carbohydrate diet is sustained (Figure 3). To our knowledge, this study is the first to report *in vitro* ethanol production by *E. clostridioformis*, *K. michiganensis, P. grossensis*, and *T. ramosa*. Additionally, this report is the first of *E. clostridioformis* growth in the presence of high levels of ethanol.

According to our findings, ethanol production varies with the species and glucose concentration in the growth medium. For instance, while *K. pneumoniae* increased ethanol production as the glucose concentration increased, *Enterocloster* decreased ethanol production under the same conditions. These results imply the influence of glucose intake on endogenous ethanol production and suggest that carbohydrate intake may play a key role in the management of these diseases.^38^

Enriched species in the intestinal microbiota of MASH patients, including yeasts, *Enterocloster* spp., *Klebsiella* spp. and *T. ramosa,* produce ethanol and hence may contribute to MASH pathogenesis. Xue et al. previously reported that reducing the abundance of ethanol-producing bacteria using antibiotics could be a novel therapeutic approach.^12^ In our study, these pathobionts demonstrated high *in vitro* susceptibility against non-absorbable antimicrobials such as amphotericin B, rifaximin, and vancomycin (FC/MIC = 15-355,000). Each of these drugs administered alone in experimental *in vivo* models improved MASH.^39-41^ In clinical practice, patients with both ABS and MASH have been successfully managed with antimicrobials, low-carbohydrate diets and probiotics (Table 2).

**Table 2.** Cases of ABS cured by antimicrobials in literature.

Clinical disease(s)	Medical history	Max BAC (g/L)	Pathobiont(s)	Antimicrobial agents	Outcome		References
**Antifungal**							
ABS	NA	4.1	*Saccharomyces cerevisiae, Candida intermedia, Klebsiella pneumoniae* and *Enterococcus faecalis*	Micafungin	Cure	BAC (0 g/L)	Saverimuttu et al. [42]
ABS	NA	4	*Saccharomyces cerevisiae, Candida albicans, Candida parapsilosis*	Micafungin	Cure	BAC (0 g/L) and no fungal growth	Malik et al. [43]
ABS	NA	4	*Saccharomyces cerevisiae*	Fluconazole then Nystatin	Cure	BAC (0 g/L) and negative stool cultures	Cordell et al. [44]
ABS	SGS	3.5	*Nakaseomyces glabratus* and *Saccharomyces cerevisiae*	Fluconazole	Cure	Symptoms resolved	Dahshan et al. [45]
ABS	NA	2.5	*Candida albicans* and *Candida krusei*	Trichomycin	Cure	Symptoms resolved	Kaji et al. (case 1) [46]
ABS	SBS	0.7	*Candida kefyr* and *Saccharomyces cerevisiae*	Fluconazole	Cure	Symptoms resolved	Jansson-Nettelbladt et al. [47]
**Antifungal and Antibacterial**							
ABS*	CIPO and SIBO	1.1	*Candida albicans* and *Saccharomyces cerevisiae*	Fluconazole and Antibiotics	Cure	BAC (0 g/L)	Spinucci et al. [48]
**Antibacterial**							
ABS	NA	4	*Klebsiella pneumoniae, Klebsiella quasipneumoniae and Klebsiella variicola*	Levofloxacin	Cure	BAC (0 g/L)	Xue et al. [12]
ABS*	NA	4	*Klebsiella pneumoniae*	Antibiotic (not detailed)	Cure	Recovery and MASH severity alleviated	Yuan et al [10]

ABS: Auto-brewery syndrome, Antibiotic: details not precise by authors, BAC: Blood alcohol concentration, CIPO: Chronic intestinal pseudo-obstruction, SBS: Short bowel syndrome, SGS: Short gut syndrome, SIBO: Small intestinal bacterial overgrowth, *: respectively MAFLD (metabolic dysfunction-associated fatty liver disease) and MASH (metabolic dysfunction-associated steatohepatitis).

The FC/MIC ratio of rifaximin was high for most bacteria, except *T. ramosa*, which showed resistance to rifampicin analogs.^49^ ^,^ ^50^ This is relevant because *T. ramosa* is associated with liver disease, colorectal cancer and genotoxicity.^17^ ^,^ ^51^ ^,^ ^52^ Alignment of *rpoB* did not clarify the resistance mechanism, and the absence of susceptible strains suggests intrinsic resistance. These findings suggest that vancomycin warrants further investigation as an alternative candidate against *T. ramosa* in future preclinical studies. However, several limitations of the FC/MIC ratio should be acknowledged. First, the FC/MIC ratio was inspired by serum pharmacokinetics and has not been validated for fecal samples, which are heterogeneous and variable across individuals.^28^ Hence, this ratio should not be overinterpreted as a clinical dosing guide, and overreliance could be dangerous in the context of antibiotic resistance. The FC/MIC >10 is therefore only an exploratory *in vitro* criterion to generate hypotheses for future preclinical studies.

In addition, antimicrobial exposure can exert selective pressure that promotes the dissemination of antimicrobial resistance genes.^53^ In this study, one of the *E. clostridioformis* genomes analyzed in our study harbored all genes belonging to the *vanB* operon, which has previously been identified in this species.^54^ ^,^ ^55^ Although Marvaud et al. reported that the *vanB* operon in *E. clostrioformis* was not transferable to *E. faecium,* ^54^ transfer to other species or under other conditions was not assessed. Thus, vancomycin resistance determinants are a limitation for any vancomycin-based strategy. Further studies, including *in vivo* models, are necessary to assess the mobilization risk and ecological impact of these resistance genes. Moreover, broad-spectrum agents like vancomycin can promote dysbiosis.^41^ Probiotics and fecal microbiota transplantation (FMT) have been proposed to mitigate dysbiosis in other contexts,^10^ ^,^ ^12^ ^,^ ^56^ ^,^ ^57^ but their combination with antibiotics in liver diseases requires dedicated preclinical and clinical investigation.

The development of microbiota-targeted approaches may be relevant for the management of patients with conditions associated with ethanol-producing gut microbes. While research on poorly absorbed antimicrobials in this field remains in its preliminary phase, it is worth noting that microbiota-targeting approaches, such as FMT, have recently been described in the treatment of Metabolic dysfunction-associated steatotic liver disease (MASLD) and ABS.^58^ This illustrates that such strategies are being explored; however, additional studies are required to assess their efficacy and safety.

A limitation of this study was the relatively small number of strains analyzed for each species. Furthermore, we did not determine the kinetics of ethanol production according to the growth phase (exponential or stationary). Future research should focus on the detailed kinetics of microbial ethanol production and their temporal dynamics. In addition, all experiments used single strains in pure culture, whereas the gut microbiota is a complex ecosystem. Ethanol production and antimicrobial susceptibility in monoculture may differ from polymicrobial conditions, and the efficacy of non-absorbable drugs may be modulated by the microbial community. Therefore, extrapolation to the human gut requires caution. Future studies should investigate these interactions using more complex models such as, co-cultures and animal models with defined polymicrobial communities. Despite these limitations, our work provides a full characterization of 33 liver disease-associated strains for the scientific community as well as their deposition in a public culture collection.

## Conclusion

Most gut microbes associated with liver diseases in this study exhibited both tolerance to ethanol and the ability to produce it. The characterization of endogenous ethanol-producing gut microbes, including *C. albicans*, *N. glabratus*, *P. kudriavzevii*, *E. clostridioformis*, *E. bolteae*, *L. fermentum*, *K. pneumoniae*, *K. michiganensis*, *T. ramosa* and *M. gnavus,* provides evidence that these species may play a significant role in liver diseases. Consequently, the gut microbiota may represent a relevant target for future therapeutic research. Furthermore, amphotericin B, rifaximin, and vancomycin together showed high *in vitro* susceptibility against all strains tested and displayed high FC/MIC ratios. However, it is noteworthy that this study is an exploratory *in vitro* study and helps to generate hypotheses for future research. No clinical use of any antibiotic is supported by our data and extensive validation in animal models and clinical trials are required before any human application.

## Supplementary Material

Supplementary Material Supplementary Data file.docx

## Data Availability

All 33 genomes of the 33 bacterial strains of species enriched in the intestinal microbiota of patients with liver diseases are available in NBCI under BIOPROJECT: PRJNA1254750, except for Q9705 and QA0666, which were previously deposited under BIOPROJECT: PRJEB76822.

## Strains availability

All 36 strains (33 associated with liver diseases and 3 associated with controls) reported in this study are publicly available and can be ordered from our microbial collection, the CSUR https://csur.eu/and by mail: contact@csur.eu. The number of each strain is reported in Table 1.

## Abbreviations

ABS: Auto-brewery Syndrome
AH: Alcoholic Hepatitis
CLSI: Clinical Laboratory Standards Institute
Cmax: Maximal concentration
COS: Columbia plus 5% Sheep Blood
EtOH: Ethanol
FC: Fecal concentration
FMT: Fecal Microbiota Transplantation
HS‒GC‒MS: Headspace gas‒gas chromatography coupled with mass spectrometry
HBV: Hepatitis B Virus
HCV: Hepatitis C Virus
MIC: Minimal Inhibitory Concentration
MASLD: Metabolic Dysfunction-Associated Steatotic Liver Disease
MASH: Metabolic Dysfunction-Associated Steatohepatitis
MAFLD: Metabolic Dysfunction-Associated Fatty Liver Disease
RRDR: Rifampin Resistance-Determining Region
rpoB: *β* subunit of bacterial RNA polymerase
